# Intranasal Administration of dsRNA Analog Poly(I:C) Induces Interferon-α Receptor-Dependent Accumulation of Antigen Experienced T Cells in the Airways

**DOI:** 10.1371/journal.pone.0051351

**Published:** 2012-12-07

**Authors:** Beth McNally, Meredith Willette, Fang Ye, Santiago Partida-Sanchez, Emilio Flaño

**Affiliations:** 1 Center for Vaccines and Immunity, The Research Institute at Nationwide Children’s Hospital, Columbus, Ohio, United States of America; 2 Center of Microbial Pathogenesis, The Research Institute at Nationwide Children’s Hospital, Columbus, Ohio, United States of America; 3 The Ohio State University College of Medicine, Columbus, Ohio, United States of America; Duke-National University of Singapore Graduate Medical School, Singapore

## Abstract

Polyriboinosinic-polyribocytoidylic acid (pIC), a synthetic dsRNA, acts as an adjuvant that boosts immune responses and protection. Intranasal (IN) administration of pIC has recently been used to adjuvant influenza virus vaccines; however, the effects of IN pIC administration on pulmonary T cell responses remain unclear. Here we show that a single IN administered dose of dsRNA into mice induced local Th1 chemokine production in the lungs and airways, and generated a biphasic and sustained migration of T lymphocytes to the airways. Furthermore, IN pIC-induced chemokine production and T cell recruitment to the airways were interferon-α receptor (IFNAR) signaling dependent. The effect of dsRNA on T cell recruitment to the airways was also dependent on the presence of high molecular weight (HMW) pIC, as a low molecular weight (LMW) pIC preparation known to only interact with TLR3 did not elicit the same effect on T cell migration to the airways, suggesting that the observed effects were dependent upon dsRNA recognition by multiple pattern recognition receptors (PPRs). IN pIC was additionally capable of stimulating low levels of T cell proliferation in the draining lymph nodes approximately 4–6 days after treatment that preceded a small population of de-novo T cells found in the airways by day 10. Taken together, these results demonstrate that the adjuvant effect of IN pIC that results in enhanced T cell proliferation and sustained T cell recruitment to the airways requires multiple PRRs and IFNAR signaling.

## Introduction

An ideal adjuvant serves to enhance the action of vaccines (thus reducing the dosage of vaccine that needs to be delivered) without inducing strong direct effects on its own. The most commonly used adjuvant, aluminum hydroxide, primarily induces a Th2 type antibody response. Yet, for infections such as tuberculosis, human immunodeficiency virus and respiratory syncytial virus (RSV) increasing evidence indicates that there exists a need for adjuvants that can additionally bolster cellular immunity. Thus, it is coming to light that adjuvants that can induce both cellular and humoral immunity are optimal. As such, agonists that trigger PRRs are currently viewed as attractive candidates for vaccine strategies [1], [2].

PRRs can sense conserved molecules specifically associated with microorganisms such as lipopolysaccharide, flagellin and microbial nucleic acids that are collectively known as pathogen-associated molecular patterns (PAMPs). PAMPS can induce immediate, innate, nonspecific immune responses as well as to enhance the efficacy of the adaptive immune response. Thus far, activation of Toll like receptor signaling (TLR) pathways by TRL agonists (PAMP or PAMP mimics) has shown success in protection against various infectious agents such as influenza virus [3], RSV [4], SARS-CoV [5], human papillomavirus [6] and hepatitis B virus [7].

The use of synthetic dsRNA as an adjuvant is a particularly interesting choice for viral adjuvant/vaccine strategies since, in addition to dsRNA viruses; nearly all viruses generate dsRNA replication intermediates. Furthermore, receptors to detect dsRNA are present on the cell surface, in the cell cytosol, and within the endosome; and are found in and on multiple cell types. PRRs that recognize dsRNA include TLR3, a membrane bound receptor found on the cell surface and in endosomes; the RNA helicases: RIG-I and melanoma differentiation-associated gene 5 (MDA-5); and the NLR pyrin domain (NLRP) 3 protein of the NLR family, the latter of which are all cytoplasmic viral RNA sensors. Ligation of these PRRs with dsRNA results in the release of inflammatory cytokines and type I interferons (IFNs), and can stimulate both innate and adaptive immune responses.

Type I IFN induced by pIC has been shown to be essential for its adjuvant effect on both humoral and cellular immunity. pIC has been shown to enhance isotype switching [8] and antibody titers, while it also appears to effect TLR3-mediated cross-priming of CD8^+^ T cells [9], [10] and CD8^+^ T cell differentiation and expansion [11], [12]. Thus far, synthetic ds RNA, pIC and polyICLC, a RNase resistant pIC analogue stabilized with poly-L lysine, have proven to be quite a successful vaccine adjuvant in mice and non-human primates [6], [13]–[15]. Recently, a systems wide analysis of single dose polyICLC in healthy human volunteers showed that the synthetic dsRNA was capable of inducing similar innate pathways as the yellow fever vaccine (as well as being well tolerated) [16]. Interest in the mucosal adjuvant effect of synthetic ds RNA has also gained popularity as studies have indicated that mucosal immunity achieved by natural infection is more effective and protective against viral infection compared to systemic immunity induced by parenteral vaccines [17], [18]. Studies using IN immunization of vaccine in combination with pIC have convincingly shown protective effects against influenza virus in mice and non-human primates [15], [19], [20].

Yet, although the potential use of dsRNA as a safe an effective adjuvant has been demonstrated in mouse and man, a complete understanding of the mechanism of action is just starting to unravel. In this study we find that IN administration of dsRNA induces Th1 chemokine production in the lungs and airways, which correlates with increased numbers of non-antigen specific CXCR3+ T lymphocytes that migrate to the lung and airways. The migration of T cells to the respiratory tract occurs in a biphasic manner and is IFNAR signaling dependent. We furthermore demonstrate that synthetic dsRNA is capable of eliciting low levels of T cell proliferation in the draining lymph nodes.

## Results

### dsRNA Induces the Production of Th1 T Lymphocyte Chemoattractants in the Lungs and Airways in an INFAR Signaling Dependent Manner

**Figure 1:**
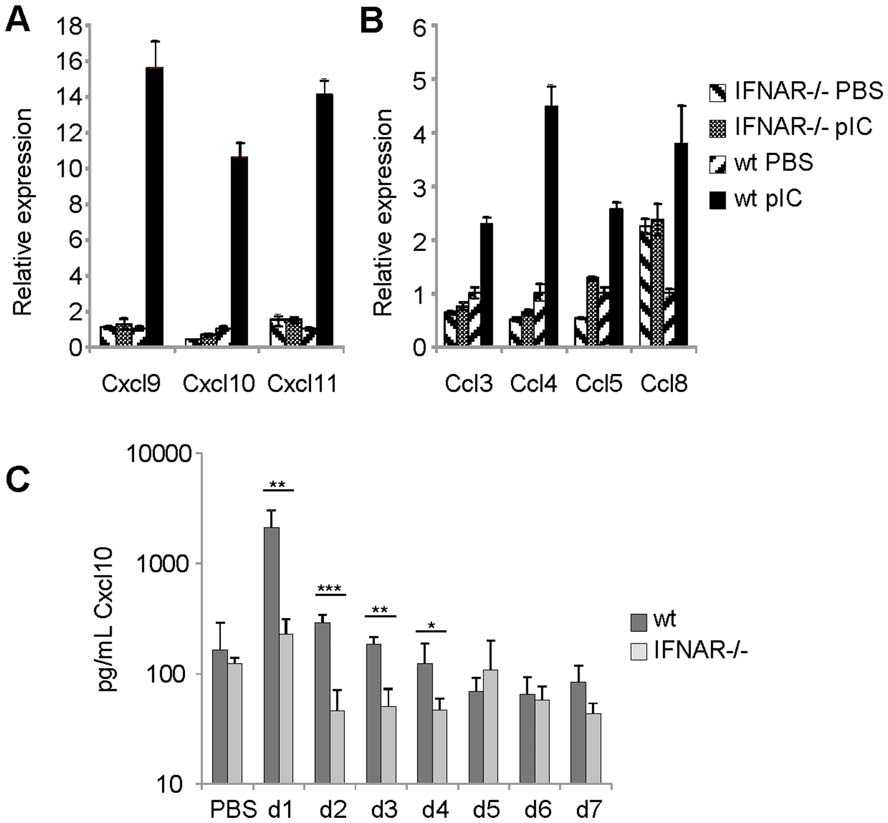
dsRNA induces IFNAR signaling dependent expression of CXCR3 ligands in the lung and airways. Total RNA was isolated from whole lung tissue of wt balb/c and IFNAR−/− mice following 48 hours of treatment with pIC or PBS (control). The data measured by Reverse Transcriptase real time-PCR represents the relative fold increase in expression of (A) CXCL9, CXCL10 and CXCL11 or (B) CCL3, CCL4, CCL5, and CCL8 using Gapdh as the endogenous control and wt PBS treated mice as the calibrator. (C) The concentration of CXCL10 in the BAL fluid of wt and IFNAR−/− mice on days 1–7 post IN treatment with pIC or PBS determined by ELISA. The data are representative from two independent experiments with 3 mice/group. Error bars represent standard deviation.

Subcutaneous injection of synthetic dsRNA has been demonstrated to enhance the production of the Th1 chemokines CXCL9 and CXCL11 in serum and in draining lymph nodes [6]. Conversely, the Th2-biased adjuvant, aluminum hydroxide, is known to inhibit TLR-induced production of CXCL10 [21]. To investigate the Th1 chemokine response to IN administered pIC, the relative expression of Th1 chemokines was measured in lung tissue via RT-qRT-PCR 48 h post treatment. The data show that the expression of the CXCR3 ligands CXCL9-11 was enhanced 10–15 fold in pIC-treated wild-type mice compared to PBS-treated controls (**Figure 1A**). Because pIC is an effective inducer type I IFN relative to other TLR-agonists [22] we also examined the role of IFNAR signaling in this process. pIC treatment failed to induce the expression of CXCL9-11 in IFNAR−/− mice. The analysis of the CCR5 ligands CCL3-5 and CCL8 also showed a similar pattern of expression (**Figure 1B**). We detected an increase in CCL3-5 and CCL8 expression in wild-type mice (2-5-fold) after pIC treatment compared to control mice. However, pIC treatment was incapable of enhancing the production of these chemokines in mice lacking signaling through the type I IFN receptor. To determine if IN pIC administration influenced Th1 chemokine protein levels present in the airways, both wild-type and IFNAR−/− mice were treated with pIC from days 1–7 and the concentration of Cxcl10 in the broncho-alveolar lavage fluid (BALF) was analyzed by ELISA. The data show that IN pIC resulted in an approximate 13-fold induction of Cxcl10 in the airways of wild type mice by 24 hours (**Figure 1C**). Additionally, the levels of Cxcl10 were approximately 9 fold greater in airways of wild-type mice 24 hours post treatment compared to IFNAR−/− mice. Furthermore, the levels of Cxcl10 in the airways of pIC-treated wild type mice remained statistically greater than the levels found in pIC-treated IFNAR−/− mice up to day 5 post treatment. These experiments indicate that IN pIC administration is sufficient to induce Th1 chemokine production in the lung parenchyma and the airways, and confirm that the expression of these chemokines is dependent on type I IFN signaling.

### IN Administration of dsRNA Promotes T Lymphocyte Recruitment to the Airways

**Figure 2:**
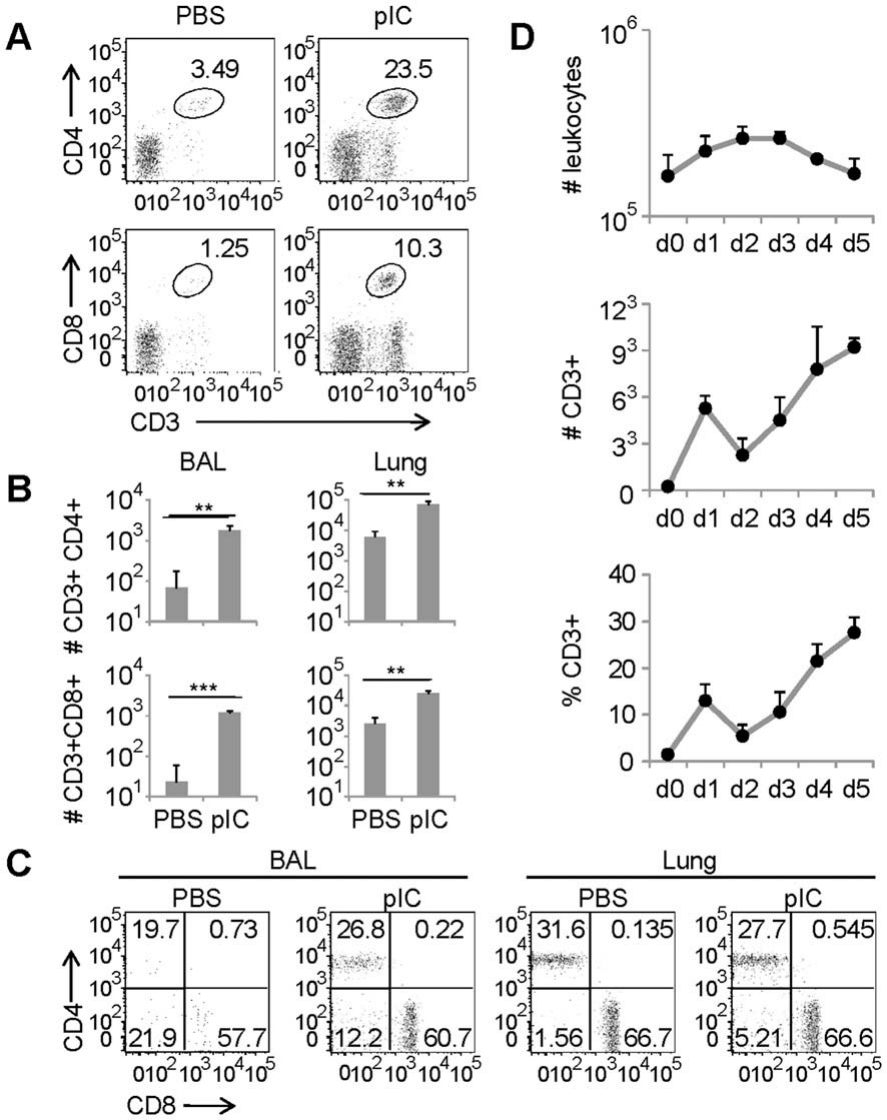
Intransal administration of dsRNA induces T cell migration into the airways. (A) Representative FACS plots show the frequency of CD4 and CD8 T lymphocytes in the BAL of Balb/c mice 2 days after IN administration of PBS or pIC. (B) Bar diagrams show the frequency of CD4 and CD8 T lymphocytes found in the BAL and lung parenchyma of balb/c mice 2 days after pIC or PBS instillation (three mice per group; representative of at least five separate experiments). **, p≤0.01; ***, p≤0.001 (C) FACS plots show the expression of CD4 and CD8 T lymphocytes within the CD3 subpopulation found in the BAL and lung parenchyma of Balb/c mice treated with or without pIC (d2). (D) Groups of 3 mice treated with pIC or PBS were harvested at the indicated time points. The absolute number of leukocytes (upper panel), the absolute number of T lymphocytes (center panel) and the frequency of T lymphocytes (bottom panel) present in the BAL after IN treatment was determined by FACS analysis. The data are representative from two independent experiments with 3 mice/group. Error bars represent standard deviation.

As Th1 chemokines are known for their ability to attract effector T cells through interactions with the CXCR3 and CCR5 receptors [23], [24] and CCR5 is necessary for early recruitment of memory T cells into the airways [25], we asked whether or not IN administration of pIC affected T lymphocyte recruitment to the lung and airways. Mice were IN treated with pIC and the frequency and absolute number of T lymphocytes present in BALF and lung parenchyma was determined 48 h later. As shown in **Figure 2**, by two days post IN pIC administration we were able to detect distinct increases in the frequency of CD4 and CD8 T lymphocytes in the BALF **(** **Figure 2A** **),** which corresponded to significant increases in the absolute number of T lymphocytes trafficking to the lung parenchyma and airways **(** **Figure 2B** **)**. Further analysis indicated that this process was due to an overall increase in T lymphocyte frequency, and not to specific changes in CD4 or CD8 subpopulations (**Figure 2C**). A temporal kinetic analysis of T lymphocytes present in the airways following pIC treatment revealed that IN pIC induced two waves of CD4 and CD8 T lymphocyte recruitment to the airways (**Figure 2D** **)**. The early (day 1) increase in T cell numbers corresponded with an increase in the number of leukocytes. Yet, over time, total leukocyte numbers in the airways decreased while there was a resurgence in the frequency and absolute number of T lymphocytes. Thus, it appears that the first wave of pIC induced T cell recruitment to the airways corresponds with inflammatory leukocyte migration while there is a specific enrichment of T cells in the second wave.

**Figure 3:**
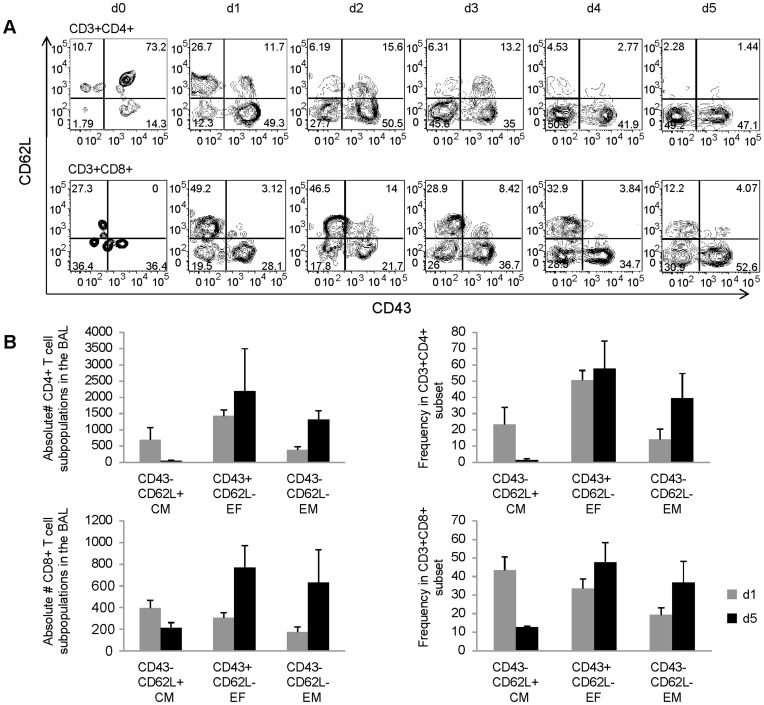
The kinetics of effector and memory T lymphocytes in the airways following pIC stimulation. (A) Representative FACS plots show the frequency of CD62L and CD43 cells present in the CD4 and CD8 T lymphocyte populations located in the BAL of balb/c mice on days 1–5 after IN administration of pIC. (B) The absolute number (left panels) and frequency (right panels) of central memory, CM (CD43^-^CD62L^+^), Effector, EF (CD43^+^CD62L^-^) and Effector memory, EM (CD43^-^CD62L^-^) CD4 (top panels) and CD8 (bottom panels) T lymphocytes in the BAL of balb/c mice on days 1 and 5 after pIC or PBS instillation.

Further characterization of airway T lymphocytes to distinguish memory and effector T cell subsets showed that central memory cells (CD62L^high^CD43^low^) constituted a fairly large fraction of the CD4 and CD8 T cells (26% and 50%, respectively) present in the airways on day 1 after pIC administration (**Figure 3**). In contrast, by day 5 after pIC treatment central memory T cells waned from the airways (2% of CD4 and 12% of CD8 T lymphocytes) and effector memory (CD62L^low^CD43^low^) and effector (CD62L^low^CD43^high^) T cells constituted the majority of the T lymphocytes present in the airways.

**Figure 4:**
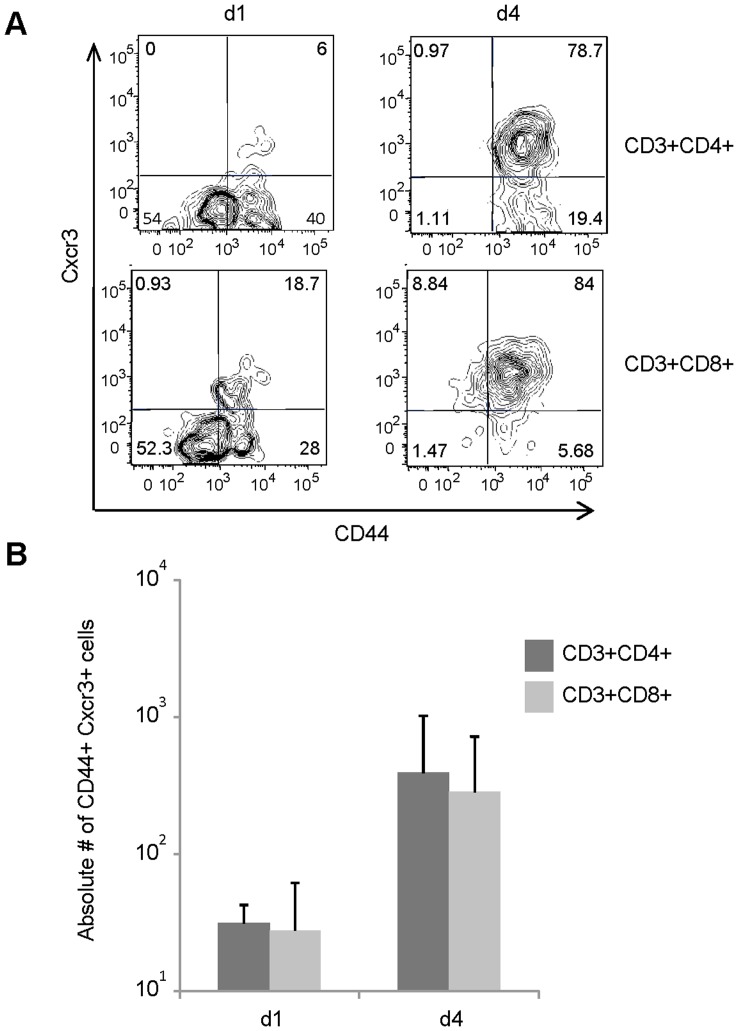
T cells recruited to the lung airways express CXCR3. Expression of CXCR3 on T lymphocytes of lung airway cells obtained from the BAL fluid of wt balb/c mice was compared on days one and four following IN administration of pIC. IN administration of PBS served as a control. (A) Representative FACS plots show the frequency of CD44 and CXCR3 expression on CD3^+^CD4^+^ (upper panels) or CD3^+^CD8^+^ (lower panels) gated lung airway cells. (B) Absolute numbers of CD44^+^CXCR3^+^ T lymphocytes present in the CD3^+^CD4^+^ (dark grey bars) and CD3^+^CD8^+^ (light grey bars) populations. The data are representative from two independent experiments with 3 mice/group. Error bars represent standard deviation.

Because our earlier data showed that both CXCR3- and CCR5-binding chemokines were expressed in the lung in response to IN pIC administration, we also investigated whether the T cells present in the airways following IN pIC treatment expressed CXCR3 and CCR5. As shown in **Figure 4**, pIC treatment increased both the frequency and absolute number of CXCR3-expressing T lymphocytes in the airways. By day four after IN pIC administration, approximately 80% of the CD4 and CD8 T lymphocytes present in the airways expressed the CXCR3 receptor. Analysis of CCR5 expression showed that 3–6% airway T cells expressed CCR5 on their surface but CCR5 could be detected intracellularly in approximately 50% of the T cells (**Figure S1**). These results are in concordance with Kohlmeier et al. [25] and indicate that CCR5 is rapidly internalized following ligand binding. Cell surface staining with CD44 also indicated that the majority of the airway CD4 and CD8 T cells were antigen experienced. Taken together, our data suggest that IN administration of dsRNA induces the recruitment of CXCR3^+^ CCR5^+^ T cells to the airways.

### IFNAR Signaling Regulates T Lymphocyte Recruitment to the Airways after IN pIC Administration

**Figure 5:**
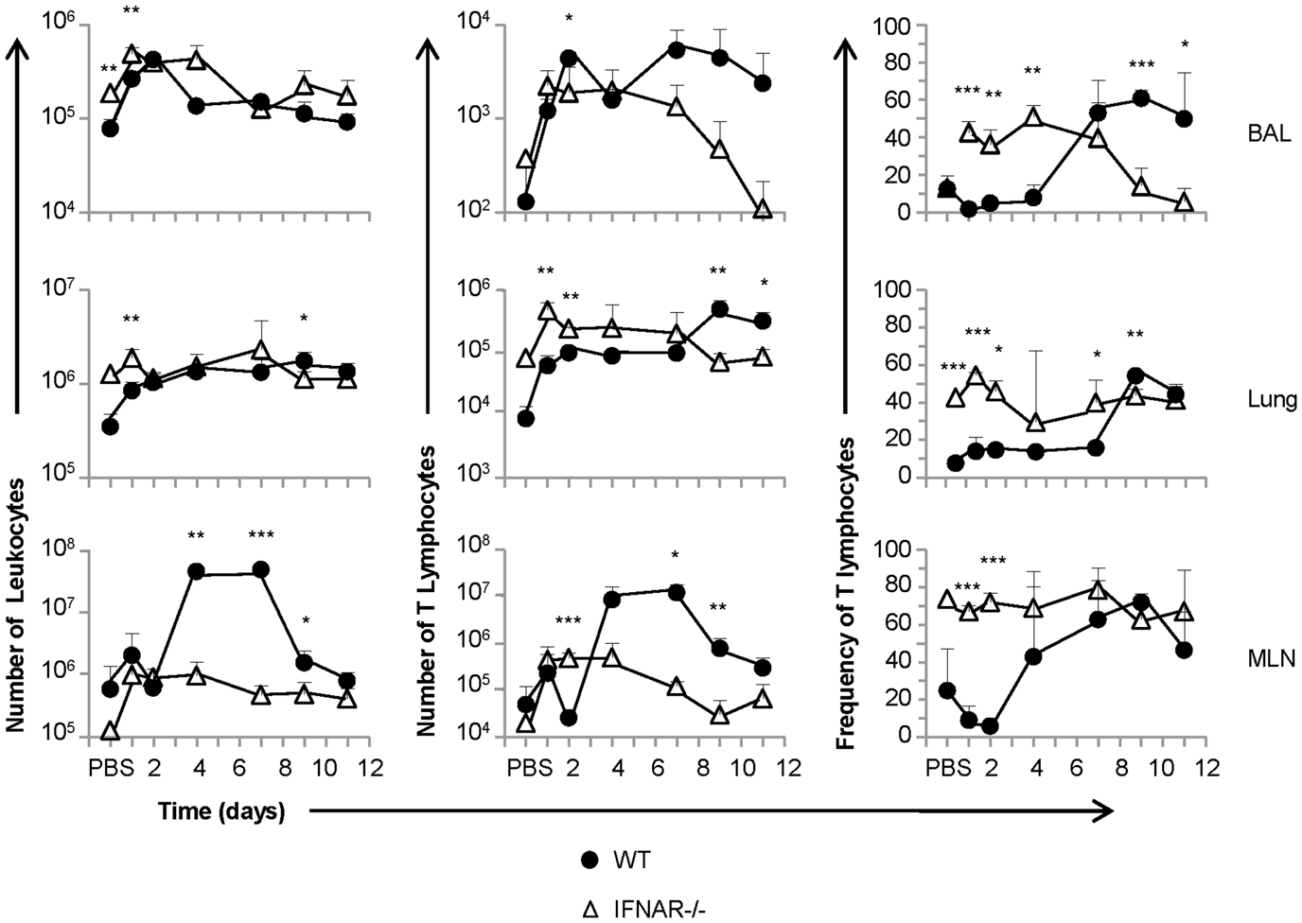
pIC induces sustained T lymphocyte recruitment to the airways that is dependent upon type I IFN signaling. Cohorts of wt balb/c and IFNAR−/− mice were treated with 50 µg pIC or PBS in an equal volume and analyzed at different time points (wt, black circles; IFNAR−/−, open triangles). The absolute numbers of leukocytes (left column); the absolute number of CD3^+^ T lymphocytes (middle column); and the frequency of CD3^+^ T lymphocytes (right column) in the BAL (upper row), lung parenchyma (middle row), and MLN (lower row) were determined by FACS analysis. The data are representative from two independent experiments with 3 mice/group. Error bars represent standard deviation. (*, p≤0.05; **, p≤0.01; ***, p≤0.001).

The findings above indicated that IFNAR signaling affected Th1 chemokine expression in the lung. Thus, we set out to determine whether type I IFN signaling could arbitrate T lymphocyte migration to the airways in response to IN pIC administration by examining pIC induced T lymphocyte recruitment in mice deficient in IFNAR signaling (IFNAR−/−). As we demonstrated earlier, IN pIC treatment induced a biphasic recruitment of T lymphocytes to the airways in wild type mice (**Figure 2D** **and** **Figure 5**). Our analysis showed that the first wave of recruitment (day 0–3) occurred both in wild-type and IFNAR−/− mice, and resulted in an overall increase in the magnitude of cellular recruitment. The second wave of T lymphocyte recruitment (day 5–12) to the airways was characterized by an increase in both the frequency and number of T lymphocytes but not in the number of mononuclear cells in the airways of wild-type mice. In contrast, the number and frequency of T lymphocytes in the airways of IFNAR−/− mice was not maintained past day 4 and declined at later time points following pIC administration. The late increase of T lymphocyte numbers in the airways was also accompanied by an increase of T lymphocyte number in the lung parenchyma and MLN in wild-type mice but not in IFNAR−/− mice. Altogether, these results indicate that the first wave of T lymphocyte recruitment into the airways was due to overall cellular infiltrate; whereas the second wave of recruitment or retention was T lymphocyte-specific and dependent on type I IFN signaling. As in vitro chemotaxis assays failed to show that wild-type T lymphocytes could migrate in response to IFNα/β gradients (**Figure S2)**, our data also indicate that downstream products of type I IFN signaling influence T lymphocyte migration rather than direct effects elicited by IFNα/β acting as a T lymphocyte chemoattractant.

### Induction of T Cell Proliferation by Synthetic ds RNA

**Figure 6:**
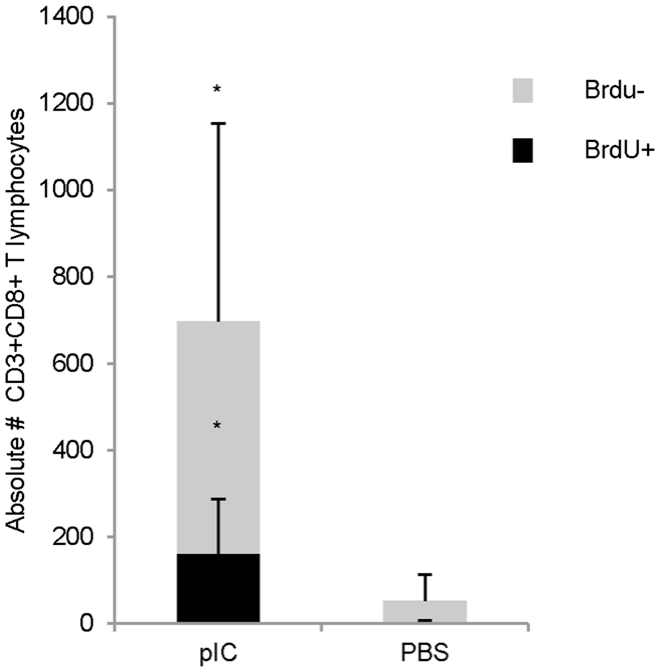
pIC arbitrates the recruitment of a small subset of de novo expanded antigen experienced cells. Balb/c mice treated with or without pIC were fed BrdU in their drinking water for 9 days and T lymphocytes found in the BAL were stained for the presence of BrdU. Bar graphs depict the number of BrdU^+^ CD8 T lymphocytes (black bars) relative to the number of BrdU^-^ CD8 T lymphocytes (grey bars). The data are representative from three independent experiments with 3 mice/group. Error bars represent standard deviation.

The data in **Figure 5** showed that in addition to the increase in T lymphocyte numbers found in the airways of wild type mice after pIC treatment, there was also an increase in the number and frequency of T lymphocytes found in the lung parenchyma and MLN. This observation suggested that increased T cell retention in the airways could not solely explain the differences observed between mice competent and deficient in IFNAR, and prompted us to ask if a *de novo* population of T lymphocytes was being generated in response to IN pIC administration. To further investigate whether or not cellular proliferation contributed to the pool of T lymphocytes trafficking to the airways in response to pIC, we examined BrdU incorporation in mice treated with or without IN pIC. The results of a time course following BrdU incorporation on days 1–7 post IN poly IC administration showed that a small percentage of CD44^hi^ T cells proliferated and peaked in the MLN of pIC treated mice around days 4–5 (**Figure S3**). By day nine, although similar levels of BrdU incorporation were found in the T cells present in the lungs and MLN of PBS and pIC treated mice (data not shown), analysis of the levels of BrdU incorporation in T cells from the BAL revealed that a small but significant fraction of airway T cells had incorporated BrdU in pIC-treated mice compared to PBS controls (**Figure 6**). Despite our observation that 10% of the airway T cells proliferated in response to pIC, our data, overall, indicate that de novo T cell proliferation does not substantially contribute to the increase in T cell frequencies and numbers observed in the airways of mice treated IN with pIC. Furthermore, although the levels of proliferating cells in the draining lymph nodes stimulated by pIC treatment mice could not account for the total increase in T lymphocyte numbers found in the lymph nodes on days 4–7 after pIC treatment, our data indicate that IN pIC treatment can stimulate the proliferation of antigen experienced T lymphocytes.

### The Molecular Size of pIC Affects T Cell Recruitment

**Figure 7:**
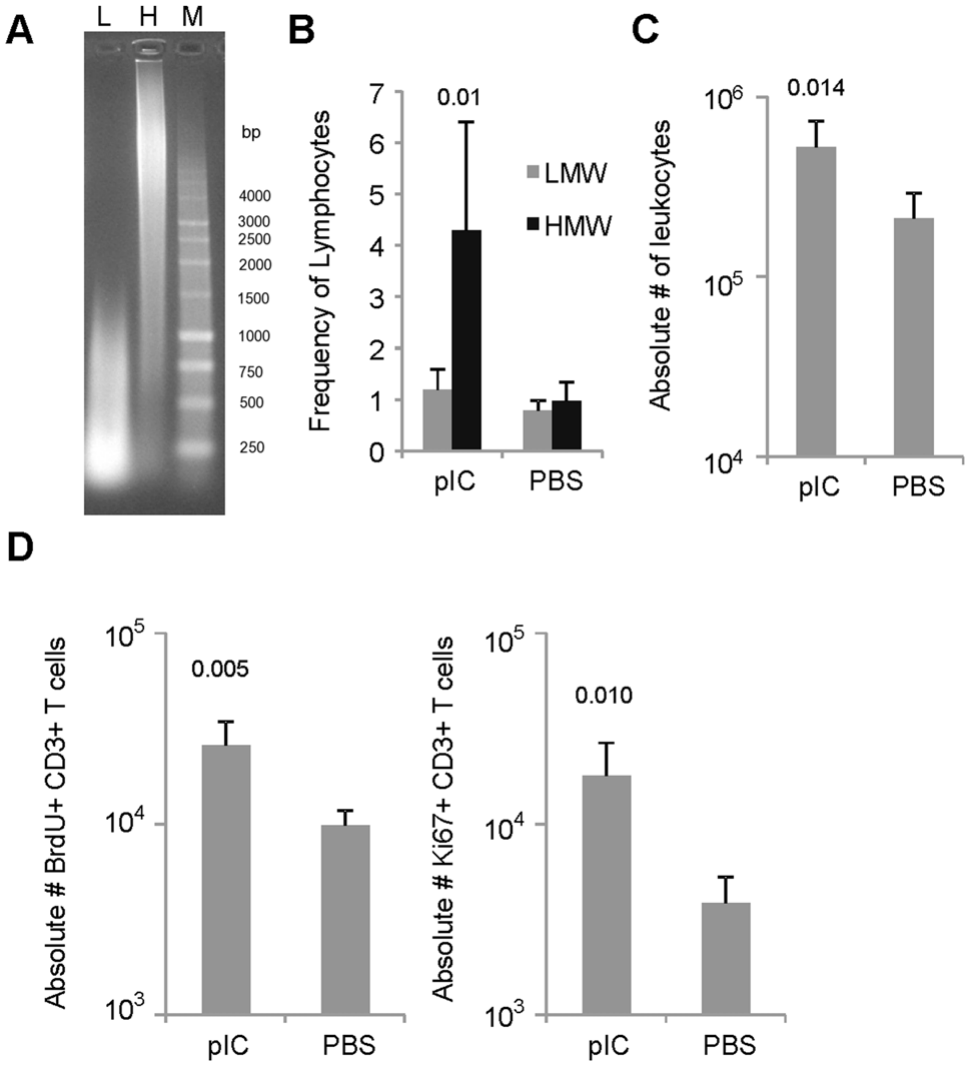
LMW pIC arbitrates recruitment of leukocytes and proliferation of T lymphocytes in the MLN. (A) Comparison of the MW of 1 µg HMW and LMW pIC loaded on a 1% agarose gel (L, LMW; H, HWM; M, 1 Kb ladder). (B) Bar diagrams generated after FACS analysis depict the frequency of lymphocytes in the BAL 9 days after IN administration of HMW or LMW pIC. (C) The absolute number of leukocytes in the MLN of mice treated with LMW pIC was determined on day 9 using an automated hematology analyzer. (D) The absolute number of proliferating T cells in the MLN on day 9 post IN treatment with LMW pIC determined by FACS analysis using a combination of intracellular and cell surface staining for BrdU/Ki67 and CD3 respectively. The data are representative from three independent experiments with 3 mice/group. Error bars represent standard deviation.

The molecular size of pIC impacts its recognition by cellular receptors and the induction of immune responses [26]–[28]. HMW pIC can signal through both cell surface and intracellular dsRNA sensors, while LMW pIC (200–1000 bps) acts exclusively through TLR3 [28]. Here we tested the effect of HMW and LMW pIC on T cell recruitment to the airways (**Figure 7**). We found that by day 9 the frequency of lymphocytes in the airways of mice treated with LMW pIC was no different than background (**Figure 7B**). However, LMW pIC significantly influenced the total number of leukocytes present in the draining LNs by d9 and the total number of BrdU^+^ and Ki67^+^ CD3 T cells in the MLN. Therefore, based on these data, it seems as if smaller chains of dsRNA which trigger TLR3 are capable of inducing proliferation of T cells in LNs, while pIC induced recruitment of T lymphocytes to the airways requires recognition of dsRNA by a combination of dsRNA PRRs.

## Discussion

pIC administration in the context of its use as an adjuvant has been shown to support the induction of both humoral and cellular immune responses [29], and recent studies support the use of IN vaccination with pIC to induce both nasal IgA and serum IgG to protect against homologous and heterologous infections [19], [20]. This study shows that IN administration of synthetic pIC in the absence of co-administered antigen can induce the recruitment of antigen experienced T cells to the airways. We also show that this result is type I IFN signaling dependent, and is only elicited by poly IC preparations that include a range of pIC chain lengths suggesting that multiple dsRNA sensors may be necessary to generate the observed effect on T cell migration.

We initially demonstrated that IN poly IC produced a Th1 response characterized by enhanced levels of Th1 relevant chemokine transcripts in the lungs and airways. Thus, by 48 h, we detected significantly greater levels of Th1 chemokine transcripts in the lung parenchymal tissue and increased levels of levels of CXCL10 protein in the airways of pIC treated mice verses PBS controls. Because CXCL10 is known for its ability to attract effector Th1 cells through interaction with its receptor CXCR3 [23], [30]–[32] we addressed the possibility that the increase in Th1 type chemokines facilitated by IN pIC promoted T cell migration into the airways. To our surprise, IN pIC resulted in two waves of T cell migration to the airways. The first wave, days 1–3 post treatment, corresponded with an overall recruitment of inflammatory cells and is in concordance with recent observations [33], whereas the second wave of T cell migration was T cell enriched and more reminiscent of an antigen driven adaptive immune response. It was possible that the effects we observed with IN pIC were a product of the route of administration, and that pIC was acting in combination with antigen washed into the airways along with the pIC. pIC in combination with antigen has been shown to not only up-regulate the expression of cytokines, chemokines and co-stimulatory molecules [13]; but to induce the proliferation of activated T cells [34]; the activation of T cells, B Cells and NK cells; the maturation of dendritic cells; and to enhance CD8 T cell cross priming [9], [10]. It is important to note, however, that antigen washed into the airways of the PBS control was not sufficient to elicit a response for T cell migration. Even more important, however, was that we were not able to replicate the effect of pIC on cytokine production and T cell migration in mice deficient in type I IFN signaling. Thus, pIC induced IFNAR signaling was essential for efficient T cell migration to the airways.

Type I IFN has been classically linked to its ability to inhibit viral replication through innate mechanisms, but IFNα/β is also responsible for modifying the adaptive immune response. Specifically, type I IFN signaling has been shown to influence T cell differentiation, survival and proliferation depending on when and how the T cell is stimulated [35] and to support CD8 clonal expansion and memory formation [36]. IFNAR signaling has also been shown to impact T cell retention in the lymph nodes [37] and T cell migration via its influence on adhesion molecules and integrins [38]. Thus, the observed differences in the numbers of T lymphocytes found in the airways of pIC treated wild-type verses IFNAR −/− mice could reflect a combination of pathways influenced by type I IFN. Although our data ultimately revealed that the numbers of proliferating cells could not account for the total numbers of T cells migrating to the airways, our results were in alignment with studies showing the effect of pIC on T cell proliferation. Tough et.al. demonstrated the effect of pIC and type I IFN on bystander T cells [39], while the combination of TLR stimulation and CD40 (also induced by pIC) has been shown to result in significant expansion of T cells [11], [39]. Thus, pIC is capable of inducing T cell proliferation in the absence of TCR recognition. Other factors such as increased T cell survival are also likely to contribute to the recruitment defect observed in the airways of pIC stimulated IFNAR−/− mice. Wiesel et. al. demonstrated that expansion of CD8 T cells is similar between WT and IFNAR−/− mice by d3 post infection but that T cell numbers dwindle thereafter in IFNAR−/− due to decreased expression of anti-apoptitic proteins in the IFNAR−/− mice [40], [41]. This data might help to explain both the similarity we found between wild-type and IFNAR−/− mice during the initial wave of T cell migration to the airways that occurred by day three post pIC treatment, and the disparity between the two groups during the second wave of T cell migration to the airways. Additionally, it is likely that the defect in IFNAR signaling contributed to poor T cell migration to the airways by negatively effecting downstream molecules such as adhesion molecules and integrins involved in cell migration and retention.

Lastly, we demonstrated that both LMW and HMW preparations of pIC were able to stimulate T cell proliferation in the draining lymph nodes. The LMW version of pIC composed of polyinosinic:polycytidylic strand lengths ranging from ∼200–1,000 bps, weekly influenced T cell migration to the airways, but significantly induced the proliferation of lymphocytes in the MLN. The HMW preparation of pIC, a mixture of strands that includes the range encompassed by the LMW version (200–10,000 bps), both induced T cell migration to the airways and T cell proliferation in the MLN. Interestingly, the LMW version of pIC is reported to exclusively act through TLR3 [28]. Thus, it is likely that TLR3 signaling was essential for the pIC induced T cell proliferation observed in the draining lymph nodes. Additionally, Avril et al. demonstrated that LMW poly IC up-regulates the CCR7 receptor on dendritic cells which is responsive to lymph node directing chemokines [28]. Although we did not specifically address other cell types influenced by pIC treatment in this study, it is very likely that dendritic cells played a large role in the observed effects. Dendritic cells express TLR3, and studies addressing the adjuvant effect of pIC have indicated that pIC induces dendritic cell maturation, activation and CTL cross-priming [27]. Furthermore, the phagocytic nature of dendritic cells might be necessary for the recognition of dsRNA by cytosolic or endocytic PRRs. Avril et al also demonstrated, using microchip analysis on dendritic cells, that HMW pIC, which is known to signal through both cell surface and Intracellular dsRNA sensors, induced a drastic up-regulation CXCL10 (IP-10), a strong attractant of activated T lymphocytes. As aforementioned in this report, we did observe enhanced levels of CXCL10 in the airways of pIC treated mice with a concomitant increase of CXCR3^+^ T cells that migrated to the airways. Thus, pIC signaling through TLR3 appears to beget proliferation of T lymphocytes, while pIC induced recruitment of T cells seems to require recognition by and signaling through multiple dsRNA PRRs.

In sum, these findings are relevant to those groups interested in vaccine strategies that incorporate the use of pIC as an adjuvant. Current studies employing pIC as an adjuvant have primarily focused on preparations that specifically act through TLR3. Our data indicates that IN treatment with HMW pIC can induce nonspecific T cell proliferation as well as sustained recruitment of T cells. This effect on T cell recruitment may be desirable under certain circumstances, but potentially damaging in instances in which strong inflammatory responses are already at play.

## Materials and Methods

### Ethics Statement

This study was carried out in strict accordance with the recommendations in the Guide for the Care and Use of Laboratory Animals of the National Institutes of Health. The protocol was approved by The Institutional Animal Care and Use Committee at the Research Institute at Nationwide Children’s Hospital (AR09-00012). All animal experiments were performed using 2,2,2-tribromoethanol anesthesia, and all efforts were made to minimize suffering.

### Mice and pIC Administration

Wild-type (wt) BALB/c mice were purchased from Harlan Laboratories, while BALB/c IFNα receptor^−/−^ (IFNAR^−/−^) mice were bred in-house. Male mice (6–12 wks of age) were IN administered 50 ug poly (I:C-HMW) (Invivogen, San Diego, California) in a 50 ul volume under light anesthesia. Organs were harvested on the indicated days post treatment, and groups of three to six animals were used for each data point. All animals were maintained in biosafety level 2 (BL2) containment under pathogen-free conditions.

### Flow Cytometry Analysis

Single-cell suspensions were obtained from the bronchial alveolar lavage (BAL), lung parenchyma, mediastinal lymph node (MLN), and spleen [42]. RBCs were lysed, and the number of cells per spleen was determined using a Sysmex counter (Mundelein, IL). Cells were stained with Fc block (CD16/32) and then washed and stained with a combination of antibodies against CD3e (clone 145-2C11), CD4 (clone GK1.5), CD8 (clone 53-6.7), CD44 (clone IM7), CD195 (clone HM-CCR5), anti mCXCR3 and mCCR5 (R&D systems), and/or CD62L (clone MEL-14), or CD43 (clone 1B11). Unless otherwise indicated, all antibodies listed were obtained from eBioscience (San Diego, CA) or Biolegend (San Diego, CA). Flow cytometry data were acquired on a BD LSR (BD Biosciences, San Jose, CA) and analyzed using FlowJo software (Tree Star, Inc. Ashland, OR). Gates were set using negative controls and isotype controls.

### ELISA

Concentrations of IP-10 (CXCL10) were measured in BAL sample supernatants using paired Abs and standards (Platinum ELISA, eBioscience). All assays were carried out according to the manufacturer’s instructions, and 100 µl from each sample was assayed in duplicate. The lower limit of detection for these assays was 7.8 pg/mL.

### Real time PCR

Total RNA was isolated from lung tissue of wt and IFNAR−/− mice by homogenizing the tissue in TRIzol (Invitrogen Life Technologies) and column purifying the aqueous phase with Qiagen’s mRNAeasy kit according to the manufactures protocol. 2 µg of total RNA was reversed transcribed in a 20 µl volume using Applied Biosystem’s high capacity reverse transcription Kit. mRNA expression was determined for 1 µl of cDNA with the PRISM 7500 sequence detection system (Applied Biosystems) using SYBR green dye and gene-specific primers designed using Primer express software. Data were normalized to glyceraldehyde-3-phosphate dehydrogenase (GAPDH) expression and the fold increase in mRNA expression was determined using the ddCt method with wt PBS treated mice as the calibrator.

### Chemotaxis Assay

In vitro chemotaxis assays were performed on purified CD8 T cells using 96-well filtration plates (Millipore) with a 5-µm pore size polycarbonate filter. 1×10^6^ T cells were added to the upper chamber containing CXCL12 (50 ng/ml) or IFNα/β (20 ng/ml) or media alone in the upper and or lower chambers as indicated. After 3 h of culture transmigrated cells were collected from the lower chamber, fixed, and counted on a flow cytometer. The absolute number of cells in each sample was determined by adding a known number of 20-µm fluorescent microbeads to each sample. Results are expressed as the mean ± SD of the chemotactic index (CI) which represents the fold increase in the number of migrated cells in response to chemoattractants over the spontaneous cell migration (to control medium).

### BrdU Administration and Staining

Balb/c mice were interperitonally (IP) injected with 1.5 mg BrdU on the day of pIC treatment and fed 0.8 mg/ml BrdU in their drinking water on the days to follow. Single-cell suspensions of the bronchial alveolar lavage, lung parenchyma, mediastinal lymph node (MLN), and spleen were prepared on day 9 after pIC treatment. Intracellular staining for BrdU and Ki67 (clone B56) was achieved using the BD Pharmingen BrdU flow kit (BD Biosciences, San Jose, CA) following the manufacturer’s instructions.

### Statistical Analysis

Statistical analysis was conducted to evaluate the significance of differences. For two-sample comparison, Student’s t-test was used to determine p values where indicated.

## Supporting Information

Figure S1 **CCR5 expression in airway T cells after synthetic dsRNA administration.** Expression of CCR5 on T lymphocytes of lung airway cells obtained from the BAL fluid of wt balb/c mice on days one and four following IN administration of 50 µg pIC (IN administration of PBS served as a control). (A) Representative FACS plots depict the frequency of CD44^+^ and cell surface CCR5^+^ T lymphocytes in CD3^+^CD4^+^ (upper panels) or CD3^+^CD8^+^ (lower panels) gated lung airway cells. (B) Absolute numbers of CD44^+^ T cells expressing cell surface CCR5 present in the CD3^+^CD4^+^ (dark grey bars) and CD3^+^CD8^+^ (light grey bars) subpopulations of lymphocytes in the airways. (C) Representative FACS plots depict the frequency of CD44^+^ T lymphocytes expressing intracellular CCR5^+^. CD3^+^CD4^+^ (upper panels) or CD3+CD8+ (lower panels) gated lung airway cells. (B) Absolute numbers of CD44^+^ T cells expressing CCR5 intracellularly present in the CD3^+^CD4^+^ (dark grey bars) and CD3^+^CD8^+^ (light grey bars) subpopulations of lymphocytes in the airways. The data are representative from two independent experiments with 3 mice/group. Error bars represent standard deviation.
(TIF)

Figure S2 **Chemotaxis of T lymphocytes to an IFNα/β gradient.** T lymphocytes isolated from spleens of naïve mice were placed in the upper chamber of a 24-transwell plate. +/− symbols represent the presence or absence of CXCL12 (50 ng/ml) or IFNα/β (20 ng/ml) in the media contained in the upper and/or lower chambers. Migrating cells were counted after 3 hours of culture and the chemotaxis index for each sample was calculated by determining the ratio of migrated T lymphocytes in chemokine-treated wells versus control wells medium alone. Results represent the means of triplicate cultures and are representative of at least three similar independent experiments. Error bars represent standard deviation.
(TIF)

Figure S3 **Kinetics of T cell proliferation in the draining lymph node following pIC treatment.** The MLNs of mice IN treated with 50 µg HMW-pIC and IP injected with 1.5 mg of BrdU were harvested on days 1–7. Representative histograms show the frequency of proliferating (BrdU positive) T lymphocytes found in the CD4^+^CD44^+^ (upper histograms) and CD8^+^CD44^+^ (bottom histograms) lymphocyte subpopulations of the MLN on days 1–7 post IN treatment with pIC. The data are representative from two independent experiments with 3 mice/group. Error bars represent standard deviation.
(TIF)
