# Supplementary figures and images for: Intranasal Administration of dsRNA Analog Poly(I:C) Induces Interferon-α Receptor-Dependent Accumulation of Antigen Experienced T Cells in the Airways
Source: PLoS One. 2012 Dec 7;7(12):e51351. doi: 10.1371/journal.pone.0051351 (PMC3517467; doi:10.1371/journal.pone.0051351)

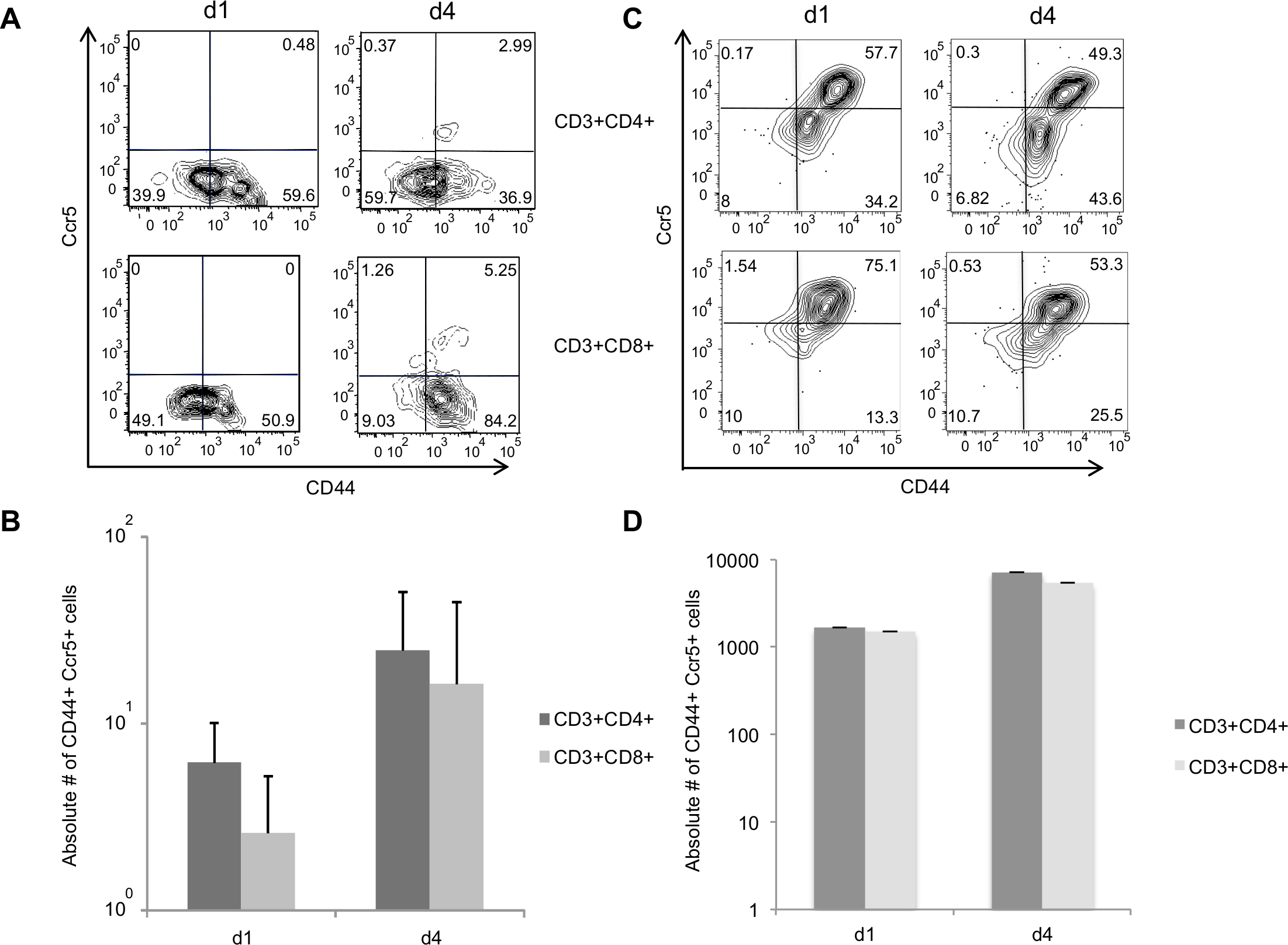

Supplement: Figure S1 — CCR5 expression in airway T cells after synthetic dsRNA administration. Expression of CCR5 on T lymphocytes of lung airway cells obtained from the BAL fluid of wt balb/c mice on days one and four following IN administration of 50 µg pIC (IN administration of PBS served as a control). (A) Representative FACS plots depict the frequency of CD44+ and cell surface CCR5+ T lymphocytes in CD3+CD4+ (upper panels) or CD3+CD8+ (lower panels) gated lung airway cells. (B) Absolute numbers of CD44+ T cells expressing cell surface CCR5 present in the CD3+CD4+ (dark grey bars) and CD3+CD8+ (light grey bars) subpopulations of lymphocytes in the airways. (C) Representative FACS plots depict the frequency of CD44+ T lymphocytes expressing intracellular CCR5+. CD3+CD4+ (upper panels) or CD3+CD8+ (lower panels) gated lung airway cells. (B) Absolute numbers of CD44+ T cells expressing CCR5 intracellularly present in the CD3+CD4+ (dark grey bars) and CD3+CD8+ (light grey bars) subpopulations of lymphocytes in the airways. The data are representative from two independent experiments with 3 mice/group. Error bars represent standard deviation. (TIF) [file pone.0051351.s001.tif]
